# Influence of Socio-Ecological and Economic Correlates on Marijuana Legalization Policy Across the States of America

**DOI:** 10.3390/ijerph22060823

**Published:** 2025-05-23

**Authors:** Mashooq Salehin, Vijayan K. Pillai

**Affiliations:** 1 Department of Social Work & Sociology, North Carolina Agricultural & Technical State University, Greensboro, NC 27411, USA; 2 School of Social Work, The University of Texas at Arlington, Arlington, TX 76019, USA; pillai@uta.edu

**Keywords:** marijuana, legalization, medical, policy, recreational, substance, United States

## Abstract

Marijuana legalization has been at the center of debate in the social and political realm of the United States. So far, thirty nine states and the District of Columbia have legalized marijuana either for recreational or medical purposes. States are taking a cautious approach to legalization in a policy context consisting of multi-layered sociopolitical systems. Strong arguments from advocacy groups supporting and opposing legalization highlight the significance of assessing the impact of marijuana use and its legalization on the well-being of the community and its members. Utilizing the social determinants of health and system perspectives, this study measures the cumulative effect of six socio-ecological and economic predictors, such as the state’s marijuana use prevalence and median household income, on the dependent variable of marijuana legality scores using discriminant analysis and One-way ANOVA. As hypothesized, the result shows a significant influence of the predictors on the state policy score of marijuana legality. Predicted associations between independent and dependent variables were also found. Findings suggest that without a supportive policy context comprising social, economic, and political factors, marijuana legalization might not have the desired impact on the community. Implications and recommendations for future research are provided.

## 1. Introduction

As the first US state, California legalized medical marijuana uses in 1996. Since then, as of March 2025, medical use of cannabis has been legalized in 39 states and the District of Columbia (DC) [1,2]. In 2012, Colorado and Washington became the first two states to legalize recreational use of marijuana through ballot initiatives; since then, as of today, a total of 24 states in addition to DC have allowed recreational marijuana use [3,4]. Marijuana legalization for medical and recreational use has gained momentums across the states in the last decades, even though federal law classifies cannabis as a “Schedule-I” substance under the Controlled Substances Act, meaning this substance with no medical use and having “a high potential for abuse” and identifies distribution of cannabis as a federal offense [1,5]. As states are taking a supportive approach to marijuana use through legalization for non-medical purposes, the issues related to production, distribution, marketing, and its impact on the health, economy, and safety of communities are becoming crucial issues in the realm of public policy at local, state and national level. Numerous reasons can be identified behind states’ preference for legalization of marijuana use, often influenced by residents’ political ideology, values, religiosity, economic or fiscal benefit, and potential positive impact on public health and crime situation [6,7]. It is important to recognize the diversity and intersection of ideologies that contribute to the complex characteristics of policies addressing marijuana use across states. This has resulted in a varied landscape concerning the definition of marijuana use for both medical and recreational purposes [8,9]. In a participatory and democratic political system, as in the United States, policies to legalize marijuana are developed in a pre-existing context comprising social, political, and economic factors. Considering the multitude of competing explanations and conjectures about the key drivers of policies, it is important to acknowledge the determinants with stronger empirical support than others. This study aims to identify the factors influencing the policies legalizing marijuana across the United States.

## 2. Policy Context

Since the beginning of the mid-1990s, states’ policy initiatives on marijuana use have adopted a cautious approach to this issue. For example, ‘decriminalizing’ cannabis use offered legal protection for the users. In general, state policy initiatives that begin with ballot measures allowing marijuana use for medical purposes are often further shaped through legislative processes that typically involve crafting and adding regulatory provisions influenced by judicial decisions. Subsequently, a more inclusive and permissive approach to marijuana use is espoused, allowing recreational use for adults typically through enacting laws with regulatory provisions to ensure the safety of the consumers and other stakeholders, such as requiring a license for the business owner and mandatory quality testing of the marijuana [2,8]. Policy analysts have noted some common approaches to marijuana legalization. Neely and Richardson [2] identified three approaches in the state policies allowing marijuana usage based on their focal points: medicalized, permissive, and fiscal. By taking a “medicalized” approach, states consider ‘medical marijuana’ as any other medication needed for the treatment of a medical condition such as pain. Policies with a permissive approach to marijuana use uphold the values of individual rights and denounce federal endorsement of marijuana as an illicit substance. State policies taking a ‘fiscal’ approach consider marijuana as a cash crop aiming to earn and/or increase revenues through taxation [2]. The common goal of reducing the economic and social cost of marijuana use is shared by all three approaches [2,7].

The socio-political context of ‘marijuana legalization’ varies from state to state; however, there is a presence of advocacy groups supporting and opposing the legalization in all states [10]. As a sensitive social and public issue, marijuana legalization has been at the center of the political debate for decades, with strong arguments from both sides. Advocates supporting legalization highlight the medical benefits of marijuana for patients with different diagnoses, a decrease in crime and expenses for relevant criminal justice services, and growth in the local/state economy through increased earning and tax revenue [5,11]. Critics of legalization argue that it triggers increased marijuana use, as well as higher rates of other drug and alcohol use, particularly among young adults [12,13,14]. They also argue that legalization negatively impacts public health, reduces traffic safety, raises crime rates, lowers educational achievement among teens [15,16], and heightens the risk of substance dependence [12,17]. However, despite strong arguments from both sides, policy analysts noted the claims of both camps as “substantially overstated” and lacking attributes of generalizability due to variations in the outcome across the states [5]. For example, although the legalization of marijuana was not found to be linked with the increase in youth usage, numerous studies reported increases in adult marijuana usage in the states where medical and/or recreational use is legalized compared to the states where such use is not legal [17,18]. Opponents of legalization underscore the assumption that relaxed marijuana laws could insinuate higher tolerance to– permitting the use of drugs, leading to increased usage of marijuana and/or other illicit drugs [17,19]. However, the findings of several studies do not fully support such an assumption. Using the data collected in the National Survey on Drug Use and Health (NSDUH), Dills et al. [5] reported mixed findings in terms of the impact of legalization on adult and youth marijuana use. Data from eight states namely, Alaska, Maine, Oregon, Massachusetts, California, Colorado, Nevada, and Washington—where recreational marijuana use was legalized between 2012 and 2016 shows a modest increase in marijuana use after legalization. It should be noted that some of these states with increased prevalence of marijuana use had a trend of increasing use even before legalization with a higher prevalence than the national average [5].

Arguments from both camps are undeniable and are based on evidence and scientific analysis. It is certainly difficult to conclude whether marijuana should be legalized for medical and/or recreational purposes since the findings of these studies are mixed. It is also important to underscore the brief time span to realize the full impact of permissive policy because it is not more than a decade and a half since Colorado and Washing first legalized recreational marijuana use. Even though states are taking time to observe the outcome of the legalization policy, based on the current trend, states are likely to take a more liberal and permissive approach to marijuana use, aiming at diverse interests and policy goals. It is observed that a state’s permissive policy influences the policy context of neighboring states, resulting in adopting similar approaches to marijuana use or being more tolerant of the usage of other substances [10,17,19]. Moreover, nationally, support for marijuana legalization has been increasing; a recent Gallup poll of November 2023 found 70 percent of respondents supporting marijuana legalization, along with all-time growing support among Republicans, with 55 percent endorsing its legality [10]. The high prevalence of marijuana usage by the US population is another crucial component of the policy context. According to the 2021 NSDUH, about 52.5 million people reported using marijuana in the past 12 months, which is almost 19% of the people aged 12 and older. More alarmingly, the survey found 8.3% of 8th graders, 19.5% of 10th graders, and 30.7% of 12th graders using cannabis in the past 12 months [13]—highlighting the risk of adolescents being addicted to alcohol and other substances resulted from using marijuana—often referred to as the “gateway of the drug,” in this developmental stage [20,21]. In the diverse multiethnic society of America, the policy initiatives of allowing the use of medical or recreational marijuana might receive mixed responses due to differences in cultural and religious perspectives on alcohol and substance use along with the influence of cultural identity, family structure, and its role [22]. This study aims to identify key social, cultural, political, and economic determinants influencing the characteristics of a state’s policies on marijuana legalization and measure their effect on those policies. The system perspective and social determinants of health (SDOH) framework have been utilized to explain the influence of socioeconomic, cultural, and political determinants on marijuana legalization policies of a state, which is discussed in the following section [23,24].

## 3. Theoretical Framework

The SDOH framework theorizes that a population would enjoy a sound social environment fostering health and well-being for its members if the determinants of the five domains facilitate supportive public policies ensuring their access to healthcare, employment, education, social and community context, and a safe living environment [25,26]. The SDOH framework underscores the influence of marijuana legalization on the social and community context of a community, as well as other social determinants that may impact the population’s health and well-being as the outcomes of the whole policy process [24,27].

The social-ecological systems (SES) theory is instrumental in illustrating the backdrop sustaining the social determinants of health in which community members interact with the socio-political, cultural, economic, and environmental systems, such as the labor market and public welfare programs at micro, mezzo, and macro levels, and share available resources to meet essential needs like food and shelter, education, employment, and healthcare [23,25,28]. Utilizing the SES perspective, the exchange of resources between interconnected and interdependent systems of individuals and surrounding environments can be explained in which micro-level factors influence the behaviors of individuals, resulting in change in the larger systems at economic, cultural, and policy settings that frame the societal laws built upon values, ideologies, and rules of human interaction [23,25,29,30]. The SES theoretical framework of the current study speculates that interrelated systems of community members and policy-making agencies, such as the state legislatures, interact and/or support each other by exchanging resources to function and maintain balance. In the process of exchanging energy or resources between systems, politically and socially sensitive issues such as ‘marijuana legalization’ may appear as a factor causing ‘entropy’ or disorder in the systems due to its perceived impact on the communities [29,30]. To minimize such threat, regulatory systems at the macro level, such as the state government, may take a restrictive or supportive approach to marijuana legalization and accordingly allocate, control, and distribute the resources through the socioeconomic systems affecting and/or benefitting individual members as the microsystems [25]. Identifying the factors influencing energy flow throughout the systems is instrumental in finding the niche within the pertinent systems to modify the policy framework regulating marijuana use. The theoretical framework of this study identifies six structural and environmental correlates based on the SDOH model to examine their influence on a state’s policy approach to marijuana legalization, representing the onset and prevalence of marijuana use by youth (12–17 years), their participation in youth and religious activities, prevalence of secondary education and household income of the states.

### 3.1. Policy Context Correlates

Propelled by the trend of marijuana legalization for medical and recreational purposes by the state legislatures, as well as increasing societal acceptance, the marijuana industry has gained significant power and a secure position through policy measures [31,32]. States’ marijuana policies reflect the extent of societal and legal acceptance as well as supportive or opposite perspectives on marijuana and its usage as such, states with permissive policies are likely to have widespread approval of this substance by the residents and their representatives in the state assembly.

The alarming prevalence of marijuana use indicates the extent of the problem and its influence on the policy climate. In general, public preferences and perceptions of an issue influence the overall political environment of a constituency, often the state, and ultimately shape the policies [33,34]. John Kingdon’s [34] policy streams model provides a conceptual framework to understand how an issue turns into a policy agenda through being recognized, and consequently seeking a solution through developing a policy [15]. The high prevalence of youth marijuana [15], well recognized as a social problem and also labeled as a substantial public health burden [32], is expected to incite the political climate of the state and create opportunities to instigate immediate and prompt solutions through policy action. According to the National Survey on Drug Use and Health [NSDUH] of 2019, about 3.3 million adolescents aged 12–17 used marijuana at least once in the previous year—about 13% of that age group; among the high school seniors, the prevalence of current users was found even higher (22%); it is estimated that each year about 1.4 million adolescents initiate marijuana use [35]. In such a context, the concern that deserves the foremost attention is the threat to adolescents’ well-being because of the spillover effect of marijuana usage on their wellness, development, and educational attainment [17,36]. Adolescents encounter various physical, social, and emotional challenges in their developmental stage, such as enjoying independence, making personal decisions, and being exposed to risky behaviors such as marijuana use. Assessing the impact of legalization through measuring the impact on adolescents and the community could be instrumental [37,38]. Hence, in the current study, we considered two predictors measuring adolescents’ (12–17 years) marijuana usage: (i) prevalence of past month marijuana use and (ii) first-time users, among the youth of (12–17 years) age group.

### 3.2. Socioeconomic Correlates

Socioeconomic correlates, such as poverty, employment, income inequality, parental marijuana use, and neighborhood environment, have been identified as the factors attributing to adolescents’ substance use, including marijuana [5]. This study uses two variables to measure the association between the state’s marijuana legalization policy and economic indicators: (i) the State’s median household income and (ii) the high school completion rate of the state’s residents.

As the indicator of socioeconomic status, the state’s median income reflects the influences of the family’s economic condition, which is associated with youth’s substance use and treatment participation [39]. The state’s median income also reflects opportunities for jobs and earnings for individuals and families and is likely to have a strong association with the educational attainment of individuals, including youth within a state, which strongly influences adolescents’ substance use, including marijuana [40].

Education as a social determinant, represented in the current study’s theoretical framework by ‘high school completion rate of the state’s residents’, denotes the extent of the youth’s access to the system of education and resources that would facilitate employment and attribute to their health and well-being [24,41]. Educated youth as individuals, are likely make better choices out of risky behaviors, such as using marijuana or alcohol at an early age, and being aware of issues affecting the community adversely, and engaging actively in social and political institutions with a view to foster a sound social and political environment for the community’s welfare [42]. Utilizing the system perspective, the current study examines the influence of social-environmental correlates of macro systems on the microsystems of young adults, indicating their opportunities to access and utilize the system of education, and measures its influence on marijuana use policy.

### 3.3. Social-Environmental Correlates

Risk and protective factors embedded in the social environment, such as opportunities for youth to participate in religious and other activities in the community, are likely to influence adolescent’s use and access to substances, including marijuana [40,43]. Religiosity has been identified as the protective factor for adolescents’ alcohol, tobacco, and substance abuse, along with delinquency and crime [44,45]. Two variables, (i) the prevalence of youth participation in religious activities and (ii) the prevalence of youth participation in youth-specific activities, were considered in the current study’s theoretical framework to measure the impact of social and environmental correlates on the state’s marijuana legalization policy.

## 4. Materials and Methods

### 4.1. Data

All fifty states of the United States were included in this study (*N* = 50). Due to its special status as a federal district under the jurisdiction of the US Congress, the District of Columbia (DC) was excluded.

#### 4.1.1. Independent Variables

Data on independent variables were collected from the National Survey on Drug Use and Health (NSDUH) restricted-use data portal (individual-level data) of 2018–2019 and were aggregated at the state level [35]. The NSDUH is conducted annually by the SAMHSA and provides nationally representative data collected from civilian, noninstitutionalized individuals aged 12 years or older on their alcohol, tobacco, illicit drugs, and other substances, including marijuana, substance use disorders, and their treatment, relevant mental health issues and use of services. This national survey collects data from the residents of households and individuals living in non-institutional settings such as shelters, boarding houses, college dormitories, and civilians living in military bases through employing a 50-state design with an independent multistage area probability sample for each state and the District of Columbia [46]. The current study used data on youth aged 12–17 years to classify adolescents based on the age group of the NSDUH category of youths [35]. Since the variable of ‘family income of the respondents’ was measured at the categorial level in NSDUH, to ensure the homogeneity of the variables’ measurement level, we used the data collected by the American Community Survey to measure median household income at the scale level [47].

To measure the effect of the predictor on the policy characteristics, this study intended to use the state policy of marijuana use in most recent years. However, since the legality of marijuana is measured based on the state’s policy in place by the year 2018, data on all independent variables except the state’s median household income were collected from the model-based prevalence estimates of the 2018–2019 NSDUH restricted-use data portal (individual-level data) of 2018–19 and were aggregated at the state level [35], [48].

Independent variables used in data analysis measures: (i) Youth’s (12–17 years) past month marijuana use that reflects the percentage of youth aged between 12 and 17 years who used marijuana at least once in the past 30 days; (ii) Youth participation in activities reflects the percentage of youth (12–17 years) who participated in two or more of the following activities: school-based, community-based, church or faith-based, or other activities such as sports in the past year; (iii) Youth participation in religious activities reflect the percentage of youth (12–17 years) who attended more than 25 religious services in the past year; (iv) Prevalence of high school completion indicates the percentage of respondents who completed high school or equivalent education among the participants of all ages residing in the respective state; (v) Prevalence of first-time marijuana users among youth reflects the percentage of youth in the age group of 12–17 years who initiated marijuana use within the past year; (vi) State’s median household income reflects the middle point of all household income, including those with no income; American Community Survey (ACS) survey data was used to measure this variable, which is a five-year (2015–19) estimate of income in 2019 inflation-adjusted dollars on the distribution of the total number of households and families, including those with no income [47].

#### 4.1.2. Dependent Variable

The dependent variable of this study is the “legality of marijuana” endorsed by a state before 2018. As explained earlier, since independent variables had data from 2018–2019, for the dependent variable, we considered the legality of marijuana on or prior to the year 2018 to measure the effect of predictors of the concurrent time period. A state received a value of 3, if both medical and recreational marijuana were legalized before the year 2018, a value of 2 was assigned to a state if only medical use of marijuana was legalized before 2018, and the state received a value of 1 in the case when neither medical or recreational use of marijuana was legalized before 2018 or, has been legalized after 2018. Data for the dependent variable were collected from multiple online sources that compiled the public records of state laws, including a database of National Conference of State Legislatures [1], Procon.org [49], and Marijuana Biz Daily [50]. The dependent variable places a state in one of the three groups noting the year of the medical and recreational use of marijuana legalization, which also denotes the extent of legality of marijuana in respective states as such, states with a value of 3 have the most permissive approach to marijuana use whereas, states with a value of 1 with most restrictive approach, and states with a value of 2 with a moderate approach to marijuana use.

## 5. Data Analysis and Results

### Figures, Tables and Schemes

A discriminant analysis (DA) was conducted as the multivariate statistical tool to assess the ability of the selected variable to predict state’s belonging to any of the three ordinal categories of marijuana legality. The dependent variable in DA, also referred to as the group variable, represents the group of a state in terms of marijuana legality endorsed by the respective state’s law. Our predictor variables were the youth’s (12–17 years) past month’s marijuana use, their participation in youth activities and religious services, the prevalence of completing high school education, median household income, and the proportion of youth who used Marijuana for the first time between the age of 12 and 17 years old. The tests of equality of group means found significant mean differences for all the predictors on the dependent variable of the legality of marijuana. Table 1 below presents the significant mean differences between the groups of states in terms of the legality of marijuana use for all independent variables based on ANOVA F statistics and the Wilks’ Lambdas. Hence, predicting the capability of group membership by the independent variables can be speculated.

Test of equality of covariance matrices tests rejected the null hypothesis of equality with a Box’s M value of 86.035 (F = 1.504, *p* < 0.05), suggesting covariance matrices do not differ between the three groups formed by the dependent variable of state’s legality of marijuana use. Discriminant analysis implemented using SPSS 24.0 yielded two discriminant functions, which aided in discriminating between categories of membership of American states on the ranking of the legality of marijuana use.

In the following step of creating discriminant functions, the DA combines the number of selected independent variables. In general, DA yields k-1 discriminant functions where k is the number of categories in the grouping variable. Two discriminant functions were identified since we have three groups in the DV. As presented in Table 2 below, the first function with a higher eigenvalue indicates higher appropriateness than the second function in predicting the legality of marijuana use among American states. The canonical correlation (which represents the multiple correlation between the predictors and the discriminant function) for the first discriminant function is 0.803, suggesting the model explains approximately 64.48% of the variation in the grouping variable, i.e., whether the use of marijuana is legal for medical and/or recreational purpose, or not legal for any purpose in a state.

The high eigen value (1.810) of the first discriminant function coupled with statistical significance of Wilks’ Lambda indicates the appropriateness of our discriminant functions. As presented in Table 3 below, Wilks’ Lambda values for both functions are statistically significant at 0.05 level, as revealed by the statistical significance of χ² statistics; hence, we conclude that both discriminant functions are significant.

Table 4 below provides each predictor’s standardized canonical discriminant function coefficient (SCC). SCC indicates the importance of the respective predictor based on its loading on the discriminant function. As presented in Table 4, “Past month marijuana use among youth” was identified as the strongest predictor, and “Median household income” was the second important predictor, followed by the variable representing the percentage of youth who used Marijuana for the first time between the ages of 12 and 17 years. With large coefficients, these three variables loading on the first discriminatory function have large values. Even though attending religious services was not identified as a strong predictor in the first discriminant function, this variable had the second largest coefficient value in the second discriminant function. However, unlike the first discriminatory function, the value was positive, suggesting association with the pro-legalization group. “Past month marijuana use among youth” was also identified as the strongest predictor in the second discriminant function. In both functions, the variable representing the percentage of first-time marijuana users among the (12–17 yr.) age group appeared as the third largest coefficient with a negative value, suggesting an association of this predictor with the groups of states that legalized marijuana use to some extent. In addition, the variable representing youth’s participation in religious services stands out with a large coefficient (1.033) as a strong predictor in the second discriminatory function.

The structure matrix presented in Table 5 below presents the structure coefficients or discriminant loading representing correlations between each variable in the model and the discriminant functions indicating the relative strength or importance of the predictors. Similar to the strength of predictors identified by the Standardized Canonical Discriminant Function Coefficients (SCC) presented in Table 4, the structure matrix presented in Table 5 also indicates the variables representing the percentage of “past month marijuana use” and, first-time marijuana users among (12–17 yr.) age group, and median household income as three of the most important predictors with large discriminant loading within the first function.

Both SCC and Structure coefficients suggest variables representing a rate of youth’s completion of high school education and participation in religious and youth activities were identified as less important or weak predictors, implying that high school education and participating in youth and religious activities are not associated with state’s legal status of marijuana. The positive values of standardized canonical coefficients suggest an association of youth’s current marijuana use with the legality of marijuana use endorsed by state law. The result of the analysis presented in Table 4 suggests that past months of marijuana use by youth and median household income are associated with state law endorsing both recreational and medical marijuana. On the other hand, the states with restrictive approaches to marijuana use are likely to have lower rates of first-time marijuana users among (12–17 yr.) age group, lower rates of high school completion, and less participation of youth in religious and youth activities.

High positive values of the standardized canonical coefficients are associated with high values of the centroids of the first discriminant function. The group centroids of the first discriminant functions for the group of states that did not legalize marijuana prior to 2018, states that legalized only medical use of marijuana prior to 2018, and the states that legalized both medical and recreational use of marijuana prior to 2018, are—1.430, 0.176, and 2.488 respectively. States with scores near to a centroid are predicted to belong to that group. To understand how efficiently the estimated discriminant functions correctly predict membership of the states of the three groups based on the legality of marijuana use endorsed by state laws, we use the classification data presented in Table 6 below.

The classification result of Table 6 reveals that overall, the proposed model correctly classified 84% of states into the groups in terms of the legal status of marijuana use by state laws. The classification analysis of Table 6 also suggests that states that legalized both medical and recreational use of marijuana before 2018 were classified with slightly better accuracy (87.5%) than states with restrictive laws that did not legalize the use of marijuana for any purpose (82.4%). The classification analysis also found that 70% of cross-validated grouped cases were correctly classified, suggesting a high level of predictive value for our model.

## 6. Discussion

The research question examined whether social determinants of health differ between the states in terms of the legality of marijuana use endorsed by the state’s law. Hypotheses of this study examined whether socio-political, demographic, and economic indicators, namely, youth’s (12–17 years) past month marijuana use, their participation in youth activities and religious services, the prevalence of completing high school education, median household income of the state, and percentage of first-time marijuana user among (12–17 yr.) age group within the state could predict the legality of marijuana use in a state. The dependent variable—marijuana legality of the state could be either restrictive, implying the use of marijuana is not legalized, or supportive, allowing only medical use of marijuana, or very supportive, allowing medical and recreational use of marijuana—which was operationalized by providing states with a score based on the legality of marijuana use of a state by 2018. The result of the data analysis suggests an association between the state’s legality of marijuana use and predictor variables. All the independent variables were identified with the predicting capability of a state’s membership to the group in terms of marijuana legality. ANOVA test revealed mean differences of all six independent variables statistically significant between states in terms of their marijuana legality. Discriminant analysis of the state’s data on socioeconomic and behavioral factors reinforced the classification of states based on their marijuana use laws. Two discriminant functions, both statistically significant, were estimated to discriminate between states in terms of their marijuana use laws. We found the state’s past month’s marijuana use as the most important predictor in both functions. In addition to the past month’s marijuana use among youth, the first discriminatory analysis identified median household income as the second most important predictor with a positive coefficient indicating that states with better economic conditions are likely to have supportive marijuana use laws or legalize marijuana use for medical and recreational use. As the third strongest predictor in both of the discriminant functions, the variable representing first-time marijuana users among (12–17 yr.) age group of the state with a negative coefficient indicates that states with restrictive marijuana use laws are likely to have a smaller number of first-time marijuana user among this (12–17 yr.) age group. We found that the legality of marijuana use endorsed by states’ laws aligns with our hypothesis that predicted states with restrictive marijuana laws are likely to have a lower prevalence of past month marijuana use by youth (12–17 yr), contrasting the states with supportive marijuana use laws. The results of the data analysis show that a state’s legality of marijuana use strongly influences the outcome of education.

The states where marijuana use is illegal for any purpose had a higher rate of respondents who completed high school education (24.98%) compared to the states that allowed marijuana use only for medical purposes (23.8%) and the states allowing marijuana for medical and recreational purpose (20.33%). ANOVA test found the mean differences in completion of high school education between the groups of states based on marijuana use law significant (F = 4.056, *p* < 0.05). Even though the variable representing education outcome of the state was not among the strong predictors, in both of the discriminant functions education was identified with a negative coefficient indicating that states with restrictive marijuana use law are likely to have a higher number of respondents with high school education. Our model supports the assumption revealing a negative correlation of past month’s marijuana use by youth with the completion of high school education by the respondents living in the state (r = −0.066), youth participation in religious (r = −0.592), and youth activities (r = −0.201). We also found a strong association between the prevalence of first-time marijuana users among (12–17 yr.) age group and the variable representing participation of youth in religious activities (r = −0.609) and youth activities (r = 0.870), indicating influence of social environment in minimizing the risk of initiating marijuana use at early age.

Our analysis also found that states with higher median household income were found to have supportive marijuana use laws. States where marijuana use is illegal for any purpose had lower median household income compared to the states where marijuana use was legal for medical and/or recreational purposes; the mean differences between the groups were statistically significant (F = 5.036, *p* < 0.05). The lower prevalence of youth participation in religious and youth activities in the states with permissive marijuana laws indicates the likelihood of having fewer options for youth activities within these states. The current model determines youth’s (12–17 years) current marijuana use, their participation in youth activities and religious services, completion of high school or equivalent education by the respondents of the state, median household income of the state, and percentage of first-time marijuana user among (12–17 yr.) age group as statistically significant predictors placing the states either in the permissive, permissive only for medical use, or restrictive group. Based on the result of the analysis, we found significant differences in the predicting variables between the “restrictive”, “moderate”, and “permissive” states with respect to their marijuana use laws.

### Limitations of the Study

There are some limitations in the study design that should be noted. The small size of the population (n = 50) is a strong factor that might reduce the ability of statistical analyses to reach statistical significance. The statistical significance was measured at the conventional 0.05 level to address this limitation. The ratio of ‘cases to independent variables’ was about 12.5:1, which is lower than the conventional ratio but adequate to prevent fatal flaws in regression analysis and might have been attributed to the low *p*-values. We collected the data on marijuana legality compiled by the National Conference of State Legislatures [1] data on cannabis legalization from the website of ProCon.org [49]—the leading source for information and research sponsored by Britannica—one of the prominent educational publishers in academia. Due to limited time and resources, we had to rely on this source for the data on the dependent variable. We used the data from the original source without any modification.

## 7. Implication and Conclusions

Underscoring the influence of sociopolitical context on the policy approach to marijuana legalization is an important implication of this study. As we theorized, based on the system and SDOH perspective, the associations between social-environmental and economic determinants and the dependent variable of the state’s legality policy score highlight the process of sharing energy or resources between societal sub-systems such as the social organization providing education, income, and opportunities for youth to participate in social and religious activities [25,29]. With a few predicting variables, this study illustrates the significant influence of social, environmental, and economic factors on the policy approach of the states to marijuana legalization. It is noteworthy to mention that the theoretical framework of the current study does not underscore any causation based on the correlations between the variables found through data analysis. The findings of this study only suggest partial aspects of the social, environmental, and economic systems of a state that predict the characteristics of the policies endorsing the legality of marijuana.

Based on the findings of the current study, authors recommend that whether legalizing marijuana would benefit the community, especially the youth or adolescents, depends on the policy, as well as on the overall socioeconomic condition providing opportunities for sustainable income, education, and engage in social, cultural and creative activities for its members. The findings of the current study illustrate the lower prevalence of adolescent marijuana in the states with restrictive marijuana laws, highlighting the impact of the implication of marijuana policy on the overall social environment of the community and, consequently, on adolescents’ health and well-being.

Whether marijuana should be legalized for recreational/medical purposes is a matter to be decided by the citizens or their representatives in the role of policymakers. The findings of this study might help policymakers make informed decisions to prioritize issues and develop or support existing programs for the well-being of young adults. For example, the findings of the current study underscore an inverse association between youth activities and marijuana use, suggesting a possible decrease in demand and use of marijuana by youth through an increase in youth participation in such activities. Allocating adequate resources for programs offering youth such activities might be an effective strategy to minimize the risk of youth using this substance in the states dealing with such concerns. Practitioners and agencies should be well informed about the possible difficulties and challenges that might come with policy change and be prepared with strategies to provide clients with the resources and services they might need.

The policy spectrum is expected to evolve in response to ever-changing public opinion. The trend of legalizing recreational marijuana, along with increasing public approval, may prompt states to develop policies that are more permissive to other recreational drugs and decriminalize substance use as an effective strategy to reduce demand and overall adverse impact on youth. Future research should focus on identifying other socioeconomic and political factors influencing marijuana legalization policies in a state. Further studies are needed to measure the impact of marijuana legalization on relevant outcomes and other health and social indicators. Qualitative studies should focus on understanding the living experiences of clients and their families resulting from the implementation of permissive and/or restrictive policies. Research is also needed to understand the influence of marijuana legalization on youth and their families of different cultures.

The dynamics of socioeconomic, political, and legal factors influencing this complex multi-layered issue of marijuana legalization are complex and deeply rooted in social, ideological, cultural, and religious values. In such a policy context, numerous challenges can be anticipated while developing and enacting a policy regulating marijuana usage. This study attempts to build a knowledge base and deepen insight into the legalization policy approach for social workers engaging in advocacy to ensure a healthy social environment for all.

## Figures and Tables

**Table 1:** Tests of Equality of Group Means.

	Wilks’ Lambda	F	df1	df2	Sig *.
Past-month adolescents (12–17 yr) marijuana use	0.519	21.79	2	47	0.000
State residents’ completion of high school education or equivalent (%)	0.853	4.05	2	47	0.024
Youth participation rate in 2 or more activities	0.879	3.23	2	47	0.048
Youth participation rate in more than 25 religious services in the past year	0.522	21.51	2	47	0.000
Median household income in the past 12 months (in 2019 inflation-adjusted dollars)	0.824	5.03	2	47	0.010
Prevalence (%) of first-time marijuana users among youth (12–17 yr)	0.630	13.81	2	47	0.000

* Significant at 0.05 level.

**Table 2:** Eigen values of discriminant functions.

Function	Eigenvalue	Percent of Variance	Canonical Correlation
1	1.810 *	85.1	0.803
2	0.317 *	14.9	0.490

* First two canonical discriminant functions were used in the analysis.

**Table 3:** Test of discriminant functions.

Test of Functions	Wilks’ Lambda	Chi-Square	df	Sig.
1 through 2	0.270	58.214	12	0.000
2	0.760	12.238	5	0.032

**Table 4:** Standardized Canonical Discriminant Function Coefficients (SCC).

Variables	Function 1	Function 2
Past month marijuana use by youth (12–17 yr.)	1.034	1.402
Prevalence of high school completion or equivalent by State residents	−0.030	−0.251
Percentage of youth’s participation in youth activities	−0.639	0.063
Percentage of youth’s participation in religious activities	−0.314	1.033
Median household income	0.730	−0.016
Percentage of first-time marijuana users among (12–17 yr.) age	−0.660	−0.614

**Table 5:** Correlations between variables and the discriminant functions.

Variables	Structure Coefficient	
	Function 1	Function 2
Percentage of past month youth (12–17 yr.) marijuana use	0.707 *	0.261
Percentage of youth’s participation in religious activities	−0.672 *	0.559
Percentage of first-time marijuana users among (12–17 yr.) age	0.570 *	−0.012
Median household income	0.342 *	−0.088
Prevalence of high school completion or equivalent	−0.300 *	−0.175
Percentage of youth’s participation in youth activities	−0.274 *	0.067

* Largest absolute correlation between each variable and any discriminant function.

**Table 6:** Classification results.

Policy Group Based on the Legality of Marijuana Use by State Law	Predicted Group Membership		
	1	2	3
Group 1: Neither medical nor recreational use of marijuana was legalized before 2018 or, has been legalized after 2018	14 (82.4%)	3 (17.6)	-
Group 2: Medical use of marijuana is legalized before 2018	2 (8%)	21 (84%)	2 (8%)
Group 3: Medical and recreational use of marijuana was legalized before 2018	-	1 (12.5%)	7 (87.5%)

84.0% of original grouped cases correctly classified.

## Data Availability

The datasets generated during and/or analyzed during the current study are available in the (i) Substance Abuse and Mental Health Services Administration [SAMHSA] repository, [https://rdas.samhsa.gov/#/survey/NSDUH-2018-2019-RD02YR] [https://www.samhsa.gov/data/sites/default/files/reports/rpt32805/2019NSDUHsaeExcelPercents/2019NSDUHsaeExcelPercents/2019NSDUHsaePercents.pdf], (accessed on 22 February 2024 )(ii) Substance Abuse and Mental Health Services Administration [SAMHSA] repository, entitled “National Survey on Drug Use and Health: 2-Year RDAS (2018 to 2019)” sponsored by the U.S. Department of Health and Human Services [https://rdas.samhsa.gov/#/survey/NSDUH-2018-2019-RD02YR], (accessed on 22 February 2024 ) and (iii) U.S. Census Bureau [https://data.census.gov/table?q=B19013:+MEDIAN+HOUSEHOLD+INCOME+IN+THE+PAST+12+MONTHS+(IN+2018+INFLATION-ADJUSTED+DOLLARS)&g=0100000US,$0400000&tid=ACSDT5Y2019.B19013&tp=true] (accessed on 23 February 2024 ).

## Abbreviations

The following abbreviations are used in this manuscript:

CBHSQ	Center for Behavioral Health Statistics and Quality
DA	Discriminant Analysis
NCSL	National Conference of State Legislatures
NIDA	National Institute on Drug Abuse
NSDUH	National Survey on Drug Use and Health
SAMHSA	Substance Abuse and Mental Health Services Administration
SDOH	Social Determinants of Health
